# The quintessence of metallomics: a harbinger of a different life science based on the periodic table of the bioelements

**DOI:** 10.1093/mtomcs/mfac051

**Published:** 2022-07-12

**Authors:** Wolfgang Maret

**Affiliations:** Metal Metabolism Group, Department of Nutritional Sciences, School of Life Course and Population Sciences, King's College London, London, UK

**Keywords:** bioelements, metallomics, periodic table

## Abstract

This year marks the 20th anniversary of the field of metallomics. As a landmark in time, it is an occasion to reflect on the past, present, and future of this integrated field of biometal sciences. A fundamental bias is one reason for having metallomics as a scientific discipline. The focus of biochemistry on the six non-metal chemical elements, collectively known with the acronym SPONCH (sulphur, phosphorus, oxygen, nitrogen, carbon, hydrogen), glosses over the fact that the lower quantities of many other elements have qualities that made them instrumental in the evolution of life and pivotal in numerous life processes. The metallome, alongside the genome, proteome, lipidome, and glycome, should be regarded as a fifth pillar of elemental—vis-à-vis molecular—building blocks in biochemistry. Metallomics as ‘global approaches to metals in the biosciences’ considers the biological significance of most chemical elements in the periodic table, not only the ones essential for life, but also the non-essential ones that are present in living matter—some at higher concentrations than the essential ones. The non-essential elements are bioactive with either positive or negative effects. Integrating the significance of many more chemical elements into the life sciences requires a transformation in learning and teaching with a focus on elemental biology in addition to molecular biology. It should include the dynamic interactions between the biosphere and the geosphere and how the human footprint is changing the ecology globally and exposing us to many additional chemical elements that become new bioelements.

## Graphical Abstract

**Graphical Abstract:**
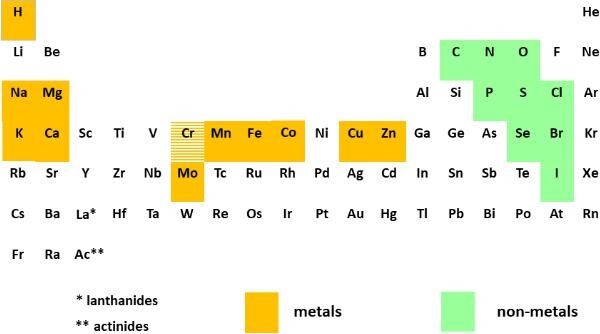
At least 20 chemical elements are essential for humans. Other forms of life have acquired yet additional elements, including metalloids, for essential functions. The total number of elements in living organisms, however, is considerably greater. Once they are present, they become *bioelements* with a plethora of beneficial or harmful functions.

## From past to present

Without metal ions life is not possible and would not have evolved in the first place. Many different metal ions have been selected for fundamental biological roles in enzyme and ribozyme catalysis, the structure and interaction of macromolecules, the regulation of metabolic and signal transduction pathways, and biomineralization. To address this metallobiology in a dedicated scientific field of collective characterization, Prof. Hiroki Haraguchi proposed the term ‘metallomics’ 20 yr ago at the international symposium ‘Bio-Trace Elements 2002 (BITREL 2002)’ in Japan.^1^ He envisioned metallomics as an integrated biometal science that complements the fields of genomics and proteomics and encompasses all scientific disciplines where metal ions have a role in biological systems.^2^ He discussed that the contemporary instrumentation for elemental analyses has the capability to detect and quantitate most of the natural chemical elements in biological material. Since elemental analyses traditionally provide total concentrations only and metals are almost always present as ions with biological ligands, which are non-metals, there is a need for element speciation to define the ‘metallome’, all metal-containing biomolecules.^3^ Thus, the analytical science underpinning metallomics also requires separation science and approaches for molecular characterization of ligands and molecules containing covalently bound bioelements—a challenging frontier in analytical chemistry.^4,5^ Professor Haraguchi also pointed out that metallomics encompasses the relationship between biochemical and geochemical processes in the environment of living organisms.

The field began to flourish and grow with seven international symposia on metallomics [(i) Nagoya, Japan 2007; (ii) Cincinnati, USA 2009; (iii) Münster, Germany 2011; (iv) Oviedo, Spain 2013; (v) Beijing, China 2015; (vi) Vienna, Austria 2017; (vii) Warsaw, Poland 2019; and this year in Japan again] and the foundation of the namesake journal in 2009.

This article discusses the scope and the significance of the field of metallomics, the challenges it faces to be recognized alongside other omics disciplines and to be integrated in such disciplines, and its relationship to other scientific fields dealing with metal ion biology such as trace element research or inorganic biochemistry. It addresses the facets of metallomics as an analytical science, a basic science to understand chemical structures and biological functions using physicochemical methods, and an applied science in terms of the synergy its translational aspects generate for fields such as nutrition, pharmacology, toxicology, and medicine. It expands on the importance of addressing the biosphere–geosphere relationship, which includes aspects of non-biological samples. Lastly, gaps in our present education will be discussed, and the need outlined for new multi- and interdisciplinary teaching and for reaching out to fields where the roles of biological metal ions are either not sufficiently appreciated or not considered at all.

### The chemical elements of life: the bioelements

To understand the significance of the chemical elements of life, one first needs to look back in time. This aspect is deemed particularly important because of the ambiguity in the meaning of some commonly employed terms such as ‘trace element’ and ‘essential’. Understanding the definitions of these terms, and in particular understanding how the presence of elements relates to functions, is crucial and a prerequisite for progress. A simple definition will be adapted: once a chemical element is present in a biological system, it is a ‘bioelement’. The initial retrospective will take a wider view about all bioelements, not just biometals, but also biometalloids (B, Si, Ge, As, Sb, Te) and non-metals, which among other functions are counterions or ligand donors of metal ions.

The development of new analytical techniques was always a prime mover in the field of biometals. Therefore, the ever-increasing detection limits for metals in chemical or instrumental analyses will serve as three milestones in the narrative. A particular statement made in the middle of the 20th century epitomizes the scientific issues lingering since then and is worth revisiting because of its importance. The late Bert L. Vallee summarized the functions of trace elements in biology:^6^ ‘Of the 98 elements of the periodic table more than sixty have been discovered in bacteria, fungi, higher plants and animals’. This statement foreshadowed the importance of many chemical elements in biological material. For the fact that they occur at high enough concentrations to be investigated with the methods available at the time, he added **magnesium** (**Mg**), **calcium** (**Ca**), **iodine** (**I**), **iron** (**Fe**), **potassium** (**K**) and **sodium** (**Na**) to the SPONCH elements, an acronym not known at the time of his writing and formulated much later for **sulphur** (**S**), **phosphorus** (**P**), **oxygen** (**O**), **nitrogen** (**N**), **carbon** (**C**), and **hydrogen** (**H**) as the six key elements of all life. **Chlorine** (**Cl**) was left out and must be added to this count. He continued:

‘Trace elements, micronutrients, microelements, minor elements and oligoelements are terms applied to the remaining elements occurring in biological systems – but in quantities so minute that no known instrumentation has allowed their measurement, and little or nothing has therefore ever been learned about their functions. As a result of this debate (about the role of the chemical elements) the term *trace element* has acquired a somewhat base and ignoble connotation, implying that these substances can only be measured qualitatively, and not quantitatively, that their presence is accidental, and therefore has no discernible consequence. This is an erroneous conclusion’.

He then discussed the advances in analytical techniques in his laboratory, in particular the use of emission spectrography, the first milestone, and focused on **copper** (**Cu**), **cobalt** (**Co**), and **manganese** (**Mn**) in his treatise. An important message here is that the term trace element is relative because it is defined based on the detection limit of the element, the ability to measure it accurately, an issue that has been long overcome while the term is still used. Vallee talked about functions of the elements, but noteworthy he did not use the term ‘essential’ for the elements present in biological material. **Zinc** (**Zn**), the element that became the focus of Vallee's research in the years to come, was not part of his discussion, though. Because zinc biology illustrates the many issues underlying the relationship between the presence of an element and its functions, it will be discussed separately. Though advances in instrumentation with ever-increasing sensitivity have contributed enormously to the field, the basic tenets of Vallee's erudite remarks still hold, and the erroneous conclusion about the presence of elements without discernible consequences still reverberates and is a key issue to be addressed.

Thirty years after the treatise of Vallee, detection limits had been lowered to the ppm and ppb levels, i.e. micrograms and nanograms/millilitre, mainly due to the advent of atomic absorption spectroscopy, the second milestone. Consequently, the term ‘ultra-trace element’ was coined for the additional elements detected and becoming amenable to investigations. While functions of **chromium** (**Cr**) and **selenium** (**Se**) in animals and humans were discussed since the 1950s, the significance of the so-called newer trace elements for animals and humans discussed since 1970 includes **vanadium** (**V**), **fluorine** (**F**), **silicon** (**Si**), **nickel** (**Ni**), **arsenic** (**As**), and **tin** (**Sn**).^7–9^ A major textbook, *Trace Elements in Human and Animal Nutrition*, lists 26 chemical elements as essential for animal and human life, 11 major elements, and 15 trace elements.^10^ The essential trace elements in animals and humans were the subject of a major review in terms of function, deficiency signs, and observed imbalances. It includes the metals V, Cr, Mo, Mn, Fe, Co, Ni, Cu, Zn, the metalloids Si and As, and the non-metals Se, F, and I.^11^ Fluorine and silicon were included because growth depression and growth stimulation were observed when these elements were restricted and supplemented, respectively. **Molybdenum** (**Mo**) was added to the elements mentioned earlier. It was known to be a constituent of xanthine oxidase since 1953.^12,13^ Classification as essential took a long time, though, because deficiency in humans is extremely rare, illustrating the point that essentiality is not always related to practical importance. In the case of some other elements, there were huge economic implications in optimizing trace element nutriture for animal husbandry and crops in agriculture, but not necessarily much impact for human health. With the lowered detection limits, a conundrum developed with conjectures remaining unresolved even today: ‘which trace elements are essential’? Some scientists maintain that an element should be considered essential only if the molecular mode of action and the structure(s) of its biological complex(es) are known. One could challenge this opinion by pointing out that elucidation of structure and function always lagged analytics, and that the statement is a case of taking absence of evidence as evidence of absence. A major issue is how ‘essentiality’ was defined and how the definition changed over time. Three criteria were put forward: ‘(i) the essential element must be present in living matter; (ii) it must be able to interact with living systems’—noteworthy both criteria apply to non-essential elements as well—and ‘(iii) a dietary deficiency must consistently result in a reduction of “a biological function” from optimal to suboptimal, preventable or reversible by physiological amounts of this element’.^14^ The earlier-mentioned criteria warranted an inclusion of fluorine because fluoride has a role in avoiding caries. Thus, inclusion in the list of essential elements becomes a matter of what is meant by ‘biological function’. The count given in 1981 was not considered final, and it may not be final in 2022. Indeed, **bromine** (**Br**) was found to be essential for humans only in the last decade,^15^ thus making all the four halogens essential, assuming fluorine deserves inclusion, if not based on dental health, perhaps on its not fully understood role in bone health. For vanadium, nickel, arsenic, and silicon, deficiency signs and imbalances had not been recorded for humans.^11^ Evidence for the postulated essentiality of these four elements and tin was summarized in chronological order in a table that includes yet additional elements: **lead** (**Pb**), **cadmium** (**Cd**), **lithium** (**Li**), and **boron** (**B**), for which positive bioactivity was reported in some experiments.^16^ Tin is a special case because only experiments in rats performed by one group are available. Except for boron, for which functional outcomes were investigated in humans, all other experimentation was performed on rats, other mammals, or chicken. How animal experiments translate to functionality in humans remains a matter of contention. The bioactivity of **aluminium** (**Al**), **germanium** (**Ge**) and **rubidium** (**Rb**) was suggested to require further investigations regarding their nutritional impact.^8^ Aluminium (Al^3+^), to which we are exposed inevitably, is mainly nowadays discussed regarding its toxic effects that are far from being understood. Its chronic toxicity has been associated with several diseases, and the controversy about the bioeffects needs to be addressed and not simply ignored.^17^ The criteria for essentiality were again modified for the discussion of the newer trace elements, which did not fulfil the original criteria of severe deficiencies being associated with growth retardation, disease, or death.^16^ Additional definitions were sought and formulated by committees. One issued by a joint endeavour of the World Health Organization (WHO/Food and Agricultural Organization (FAO)/International Atomic Energy Agency (IAEA) states: ‘An element is considered essential to an organism when reduction of its exposure below a certain limit results consistently in a reduction in a physiological important function, or when the element is an integral part of an organic structure performing a vital function in the organism’.^18^ A review entitled ‘The scientific basis for establishing the essentiality of trace elements’ emphasizes that a physiological, not a biochemical function is needed for the definition of essentiality.^19^ Indeed, often discovery of a biochemical function in an enzyme preceded that of a physiological function. Again, the term ‘important physiological function’ was not defined. The work on the additional trace and ultra-trace elements with positive bioactivity continued with the dilemma of functional significance for human health yet to be resolved in a plethora of published experiments. Even when essentiality can be shown in animal experiments, corresponding experiments in humans face the limitations that either deficiency states are not being encountered under normal conditions or performance of experiments to induce deficiency are unethical. Notwithstanding the general lack of agreement what ‘essential’ means, it was then suggested to distinguish some elements that are merely ‘beneficial’ from those that are truly essential for survival, growth, development, and reproduction.^20^ B, Ni, Si, and V fall into this category of ‘beneficial’, though they are essential for other organisms. Like the term essential, the term beneficial needs to be specified in terms of the importance of function.

The third milestone in instrumental analytical techniques was the advent of elemental mass spectrometry (MS), particularly inductively coupled plasma MS (ICP–MS). It lowered the detection limits even further to the ppt (pg/mL) range and demonstrated that many more elements are present in biological tissues. In essence, most of the natural elements now could be measured.^21^ Hence, they became bioelements. It blurred the line between accidental and constitutive presence of elements even further and amplified the question of function: What are the consequences of all these additional, apparently non-essential elements being present? It became apparent that one needs to look at the entire dose–response curve for discussing function. There is no case where an element has only one single molecular function. Elements have a range of molecular interactions and functions depending on their concentrations. Their bioactivity is further modulated by the presence of antagonists (‘antinutrients’) or agonists, both of which can be other elements, endogenous substances, or substances such as nutrients taken up from the environment. How do molecular functions then relate to systemic function? To distinguish the constitutive presence of elements from their accidental presence due to exposures, one needs to know what their ‘normal’ ranges are. It is not a straightforward endeavour as the history of lead exposure shows. Natural and contaminated lead levels are clearly different as human activities have increased lead levels in the environment.^22^ The importance of relating concentrations to functional outcomes was already formulated 500 yr ago and a reminder of the principle may be helpful. Paracelsus (Theophrastus von Hohenheim) stated that it is the dose that makes the difference between a substance being a drug or a toxicant. Supraphysiological doses of both essential and non-essential elements can elicit pharmacological effects, and even higher doses express toxic effects. Essential trace elements have toxic effects at high concentrations. Meanwhile the molecular mechanisms by which they cause cell death are becoming known. Ferroptosis, a specific term for cell death induced by an excess of iron is rather well established. Excess of copper and zinc also induces cell death. While the term cuproptosis has been introduced for a specific process of cell death triggered by copper binding to lipoylated proteins of the tricarboxylic acid cycle, a corresponding term for zinc, ‘zincoptosis’, has yet to be defined.^23^ Thus, ‘essential’ or ‘toxic’ is not an inherent property of the element but rather a consequence of its concentration in a living organism. Nutritional essentiality is difficult to demonstrate if our diet habits satisfy the requirement. With reference to the newer trace elements, the question remains, iconoclastic as may sound, whether substances that are considered to have mainly toxic effects (e.g. As, Cd, Pb) also have positive effects at lower concentrations as some experiments indeed seem to suggest. A possible resolution of the dilemma of function is that one should put less emphasis on the question whether a chemical element is essential or non-essential because a non-essential element being present can have as serious an effect as an essential one not being present. Instead, we need a different view, namely understanding functions of chemical elements over a range of concentrations. The range between essentiality and toxicity is narrow for some elements. Like vitamins, together with which the trace elements are often discussed in terms of depending on them in our diet, we need to have the right amounts. The amounts vary over at least three orders of magnitude from 18 mg iron for adult females to 2.4 μg vitamin B_12_ (cobalt) recommended per day. Definition of the right amount is extensively discussed for the elements which we believe to be essential while little or no attention is paid to whether we surpass the amounts of many non-essential elements that are clearly not non-functional either when present constitutively or accidentally. We know the negative health and disease-causing effects in some instances, e.g. Pb, Cd, As, but there is no routine monitoring of ambient exposures. Analyses are performed only when a toxic exposure is suspected or documented.

**Fig. 1:**
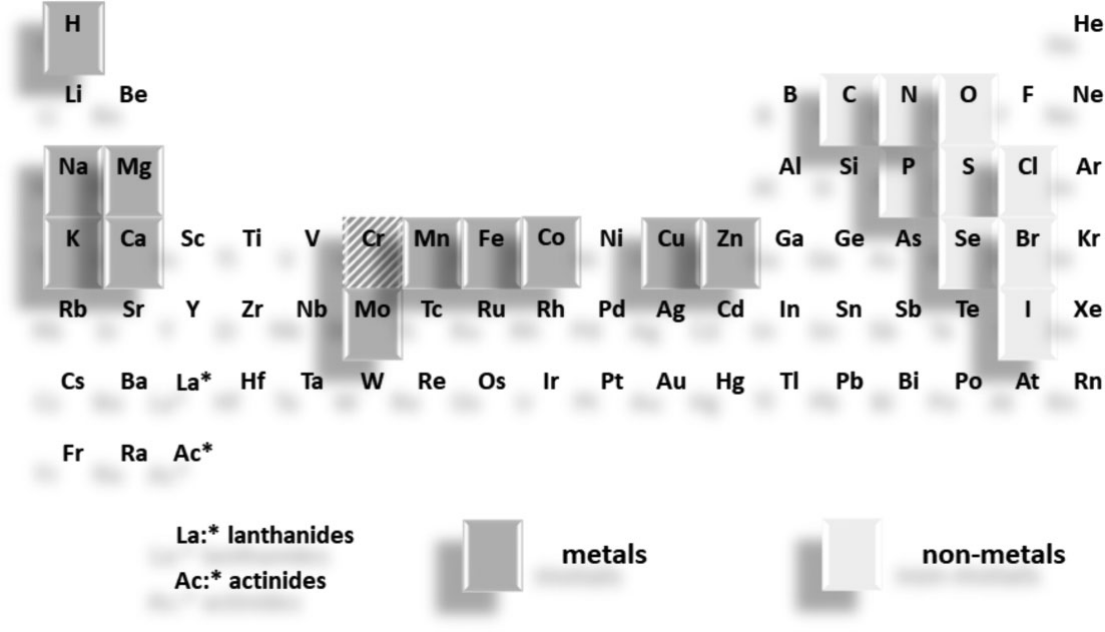
Periodic table of the chemical elements that are nutritionally essential for humans. Chromium is shaded due to the controversies about its essentiality as discussed in the main text. Figure was adapted from W. Maret, Metallomics: The Science of Biometals and Biometalloids, chapter 1 in Metallomics, M. A. Z. Arruda, ed, Advances in Experimental Medicine and Biology 1055, 1–20, 2018 with permission of Springer International Publishing AG.

A critical point in this discussion is to which group of living organisms the functional aspects such as essentiality are applied. Thus, in addition to ‘essential for which function?’ one needs to ask: ‘essential for which species?’ Periodic tables of the chemical elements required for life often include all the elements observed for functions in the tree of life. Since this approach generates considerable confusion, a periodic table of the bioelements that are essential for humans is presented (Fig. 1). It contains 20 elements to be proven essential for humans, includes chromium despite of uncertainties, and excludes merely beneficial elements.^24^ We do not know whether there are differences between humans and animals or among animals. Periodic tables for plants and microorganisms are yet different. Plants require **boron** and **silicon.**^25^**Vanadium** and **nickel** enzymes were discovered in some organisms.^26,27^**Tungsten** (**W**) enzymes and enzymes that utilize **lanthanum** (**La**) and **cerium** (**Ce**) instead of calcium have been identified in bacteria, and a **cadmium**-containing carbonic anhydrase was characterized in marine diatoms.^28–30^

Understanding which elements are essential and which biological functions the additional elements have is an important issue for human, animal, and plant health. It is far from being resolved. The answer is that we do not have all the answers. It is a stern warning to the waging nutrition debates and a wake-up call to any dietician: it is remarkable that so little emphasis is placed on the significance and often potent actions of the additional chemical elements from all standpoints: essentiality, beneficialness, or interference and interactions with other nutrients. Do we need dietary intake recommendations for beneficial elements?^20^ And should we even have intake recommendations for limiting the presence of other elements? In the realm of precision nutrition, the precise actions of the additional micronutrients discussed here and yet others thought be non-essential need to be considered, and bioelements need to be either measured directly or their status assessed through biomarkers.

### The realm of metalloproteomes

Metal ions have been discussed primarily regarding their interactions with proteins. Two approaches have been applied to characterize metalloproteomes. One is a bioinformatics approach. It employs sequence motifs (signatures) with characteristic ligands and number of amino acids between the ligands, ‘spacers’, and information about 3D and domain structures of proteins for mining entire genomes for metal-binding sites. The approach has been enormously successful for predicting the number of zinc, copper, and iron proteins in the three domains of life.^31^ There are several limitations, however. The zinc, copper, and iron proteomes are based on known sequence motifs only and such motifs are not always unique.^32,33^ A few motifs thought to be zinc-binding motifs turned out to be iron-binding sites.^34^ So, far, it has not been possible to predict the manganese proteome, apparently because of uncertainties whether manganese-binding sites have unique sequence motifs. Likewise, for non-essential metal ions, we do not have unique sequence motifs, enough information about their cellular metabolism, and their binding preferences. Importantly, not only the selectivity of the protein but the entire metal metabolism of the cell determines metal ion utilization as has been expressed by the sentence, ‘the cell rules’.^35^ The second approach is an experimental one using high-throughput tandem MS (HT–MS/MS) and ICP–MS of fractions chromatographically separated from the cytosol. When applied to the microorganism *Pyrococcus furiosus*, a hyperthermophilic archaea, it was concluded that metalloproteomes are far from being fully characterized.^36^ Among the 343 metal peaks identified in the chromatograms, 158 could not be assigned to any known metalloprotein. A total of 83 of these unassigned peaks contained metals that were known to be used by the organism: cobalt, iron, nickel, tungsten, and zinc. It came at a surprise that the remaining 75 peak fractions contained metals that the organism was not expected to accumulate: lead, manganese, molybdenum, uranium, and vanadium. In the process of characterization, four new nickel and molybdenum proteins were found. Four purified proteins contained sub-stoichiometric amounts of lead and uranium. The assimilation of ‘unexpected’ elements was confirmed in two other microorganisms, demonstrating the presence of elements with unknown functionality. These observations are not only important for an appreciation of the many bioelements in biochemistry, but also for considering unexpected micronutrients that we obtain from plant and animal food while also hinting to growth requirements of our microbiota and their largely uncharacterized and complex metalloproteomes.

### Zinc as an example for the universal importance of a ‘trace’

Monographs can be written about the role of every bioelement and many have been written. In each case, the science is different and developed differently and often independently. **Zinc** will serve as a paradigm of the generally asynchronous discoveries in various fields and the time it took before some consensus about nutritional and biochemical significance developed.^37^

In 1869, Jules Raulin, a student of Louis Pasteur, demonstrated that zinc is essential for the growth of the fungus *Aspergillus niger*.^38^ Even more than 50 yr later, it was speculated that zinc in the human body is not a contaminant but present for a purpose.^39^ In 1934, its essentiality for rats was proved.^40^ It was not until 1961 that its essentiality for humans was discovered in a malnutrition syndrome of adolescents in Egypt and Iran, and not until 1972—100 yr after the original discovery of Raulin—that the significance for paediatric medicine in the Western world became apparent.^41,42^ However, evidence for zinc being a constituent of carbonic anhydrase existed already in 1939, almost 30 yr before the nutritional role in humans was established.^43^ And, thereafter, the number of zinc enzymes and proteins discovered increased continuously. The characterization of zinc proteins culminated in a remarkable count when sequences of entire genomes became available and were ‘mined’ for potential zinc-binding sites in proteins. It turned out that humans have about 3000 zinc proteins, meaning that every 10th protein contains zinc, and demonstrating the widespread use and significance of zinc as a catalytic, structural, and regulatory cofactor.^44^ Given the overall importance of zinc in so many processes, it does not surprise that a very complex system for the control of cellular homeostasis has evolved. It encompasses at least 24 membrane transporters and a dozen metallothioneins.^45^ The proteins responsible for cellular zinc homeostasis have many mutations that affect their function.^46^ Hence, our health depends on proper functioning of these proteins and not only on supply of zinc in the diet. Furthermore, zinc ions were recognized as signalling ions akin to calcium ions with yet to be fully explored implications.^47^ But even with this extensive biochemistry now established, the impact on medical practice remains remarkably limited, albeit zinc supplementation is a very effective means worldwide to decrease child mortality. This short history shows that zinc emerged from obscurity as one of the major elements of life. It is considered a type 2 nutrient, which has pleiotropic functions such as magnesium, in contrast to iron, which has specific functions and therefore is a type 1 nutrient.^48^

As a result of these developments several myths prevail. It is often stated that there is no storage of zinc. Yet, there are reservoirs of zinc in cellular vesicles/organelles and in metallothionein. Metallothioneins work by buffering zinc ions, and they are highly regulated themselves.^49^ Their modes of actions are optimized for control of zinc with a specific pH and redox dependence of metal binding. Zinc itself is redox-inert in biology. However, the sulphur donors of the cysteine ligands in metallothioneins confer redox activity with the consequence that zinc metabolism and redox metabolism are linked.^50^ With its redox-inert nature, Zn(II) is more similar to Mg(II) and Ca(II) in biology rather than to the redox-active transition metal ions. Flexibility in coordination and binding strength are key characteristics. Zinc is not a transition metal per definition of IUPAC, it is not an antioxidant per se as it can also have oxidant functions depending on its concentrations, and whether it is a trace element is a matter of definition as addressed in the following section.

### Quantity

Biochemistry focuses on the molecules made from the SPONCH elements, which are all non-metals, with the proviso that hydrogen is in the same group as the alkali metals in the periodic table and indeed has metallic properties under very high pressures.^51^ Living systems evolved from an inorganic world and in the process of evolution many more elements from most groups were selected, apart from groups 3 and 4, and 13 (except for boron) and group 18, the noble gases.^52^ The SPONCH elements make up the majority of biomass in a human (98%), with calcium adding 1.5%, sodium, chloride, potassium, and magnesium together adding about another <1%, thus amounting to >99%. The remaining 0.1% is made up by all the other elements that are referred to as trace elements (Table 1). Definitions of terms are not unequivocal. Where should one draw the lines for bulk elements, macro- and microminerals, and trace and ultra-trace elements in the virtual continuum of quantities of the different bioelements? Should we consider only ONCH as the bulk elements, because phosphorus and sulphur together make up only 1.3%? The SPONCH elements also do not include potassium and calcium, which occur at higher amounts than sulphur. Ca, P, K, S, Na, Cl, and Mg are often called macrominerals.

**Table 1.** Elemental composition of the human body^53^

Element	Percentage of mass
Oxygen (O)	65
Carbon (C)	18.5
Hydrogen (H)	10
Nitrogen (N)	3.2
Calcium (Ca)	1.5
Phosphorus (P)	1.0
Potassium (K)	0.4
Sulphur (S)	0.3
Sodium (Na)	0.2
Chlorine (Cl)	0.2
Magnesium (Mg)	0.1
Trace elements	<0.1

The term ‘trace element’ originated from the initial difficulties in quantitation as discussed. It is certainly problematic when used in the context of function to imply less importance. Lower quantities do not mean less quality. In nutrition, the term refers to the need of only milligrams in our diet, e.g. about 10 mg of zinc versus a recommended 1.5 g of salt (NaCl).^9^ Thus, this definition in nutrition is a consequence of the small amount required daily. In accordance with the data in Table 1, in geochemistry, chemical elements with an abundance of 0.1 weight percentage are considered trace elements. According to this definition even magnesium would be a trace element. When trace elements function as catalysts, it is the very nature of a catalyst to be active at very low concentrations. One can maintain that iron in blood is not a trace, perceptible even with the naked eye as the pigment of blood. Likewise zinc in cells is not a trace although it is invisible as zinc compounds have no colour unless a ligand confers it. Cellular zinc concentrations are about 0.2 mM or higher, almost as high as those of ATP, which is certainly not considered a trace. Together with magnesium, iron, and zinc are at the g-level in humans (see following text). And remarkably, there are two other elements with similar amounts: fluorine and silicon, both considered non-essential.

Within the group of 0.1% (‘trace elements’), the concentrations of bioelements vary over six orders of magnitude, ranging from gram to microgram. The issue of quantity is confusing for the term ‘trace element’ as opposed to ‘mineral’. Calcium and magnesium are not so much different from iron and zinc and yet the latter are often referred to as trace elements while the former are considered minerals. In terms of the overall amounts, bioelements follow the series given in Table 2.^54^

**Table 2.** Overall amount of bioelements in a human body

kg level: oxygen (43), carbon (16), hydrogen (7), nitrogen (1.8), calcium (1)
g level: phosphorous (780), potassium (140), sulphur (140), sodium (100), chlorine (95), magnesium (19), iron (4.2), **fluorine** (**2.6**), zinc (2.3), **silicon** (**1**)
mg level: **rubidium** (**680**), **strontium** (**320**), bromine (260), **lead** (**120**), copper (72), **aluminium** (**60**), **cadmium** (**60**), **cerium** (**50**), **barium** (**22**), iodine (20), **tin** (**20**), **titanium** (**20**), **boron** (**18**), **nickel** (**15**), selenium (15), chromium (14), manganese (12), **arsenic** (**7**), **lithium** (**7**), **caesium** (**6**), **mercury** (**6**), **germanium** (**5**), molybdenum (5), cobalt (3), **antimony** (**2**), **silver** (**2**), **niobium** (**1.5**), **zirconium** (**1**)
μg level (examples only): **gallium, tellurium** (**800**), **lanthanum** (**700**), **vanadium, uranium** (**100**), **tungsten** (**20**)

For the bioelements shown in bold in Table 2, essentiality for humans has not been established. Remarkably, at the milligram level, at least 16 elements that are thought to be non-essential are present at higher amounts than the two essential ones: molybdenum and cobalt. Large differences exist among the most widely discussed four essential elements in the 3d series. There are a few grams of Fe and Zn whereas there are only 72 and 12 mg of copper and manganese, respectively. In the case of manganese, it amounts to the weight of a droplet of water. The same trend [Fe], [Zn] >> [Cu] > [Mn] is observed in tissues (Table 3). With some variation, the relative ratios of concentrations are maintained in major organs. However, the concentrations are different from those in blood plasma where the ratio of iron: copper: zinc is 1:1:1, and manganese remains low (Table 3). These metals and others are often referred to as ‘heavy metals’. The term is problematic in biology as the definition is based on the density of the metal, the element, which has almost no significance for biology. There is an exception, however. Recent studies provide evidence for the presence of elemental iron and copper in amyloid plaques in the brain of Alzheimer disease patients.^55^

**Table 3.** Concentrations^a^ of the manganese, iron, copper, and zinc and their ratios in human tissues and blood plasma^57^

Metal:	Mn	Fe	Cu	Zn	Ratio (all)	Ratio (Cu/Zn)
Tissue:						
Liver	138	16 769	882	5543	1:121:6:40	1:6.3
	130–150	11 000–22 000	/	/		
Heart	27	5530	350	2772	1:204:13:103	1:7.9
	21–47	4900–7200	240–410	1635–3400		
Kidney	79	7168	379	5018	1:91:5:64	1:13.2
	40–91	6440–7700	/	2873–8100		
Pancreas	102	4633	180	2740	1:45:2:27	1:13.5
	73–110	3500–5200	90–260	2010–3600		
Lung	29	24 976	220	1470	1:861:8:51	1:6.7
	20–51	14 000–29 000	130–420	990–2200		
Brain	22	4100	401	915	1:186:18:42	1:2.3
	15–34	3400–5800	340–460	800–1200		
Blood	<0.068	1.10	1.12	1.14	1:1:1	1:1
(plasma)		0.71–1.27	0.61–1.41	0.79–1.70	(Fe/Cu/Zn)	

^a^The values (mean with the range) are in μg/g (ppm) in ashed tissue and for blood plasma in mg/L.

Metal ions have quite potent actions at low concentrations. The presence of all these elements considered non-essential raises the issue what their functions are and in which range of concentrations they can be present safely. It becomes a pressing issue as there is now precedence for a much wider variation in their presence due to human activities that increase exposures. There is a lack of biochemical systems that control them and there is no system of detoxification for dealing with the emerging exposure to elemental toxicants. How do these non-essential elements interfere with the functions of the essential metals, in particular Mn, Fe, Cu, and Zn?

The total amounts and concentrations (Tables 2 and 3) are averages over the entire human body/tissue. Metal imaging technology is an important breakthrough to map the distribution of bioelements. It is providing ample evidence for heterogeneity in distribution. Biological systems, therefore, need to be in place for establishing uneven distribution for essential metal ions. Non-essential metal ions, which can piggyback on some of the system for metal transport, also affect tissues, cell types, and organelles differently. Some elements accumulate in bone. Thus, the series for the relative abundance of elements will differ for hard (mineralized) and soft tissue. Furthermore, significant differences between extracellular, e.g. blood plasma, and intracellular concentrations of elements exist (Table 3). The concentrations of many essential elements in cells are increased relative to blood. Not only do tissues differ in their elemental composition, but also cell types and subcellular organelles. For example, iron is preferentially transported to mitochondria, which need it. To express specificity in their cellular actions and to avoid interference among elements, elements are kept at relative constant cellular ratios that are different from those in blood. Some investigators convert concentrations into the number of metal ions/cell. Such a conversion can be problematic when the low concentrations and the low volumes of cells or cellular organelles result in values that suggest less than one metal ion/unit of volume.^56^

Quantitative information about the amounts and concentrations of bioelements is essential for planning experiments.

### Essential biometals and their regulation

**Fig. 2:**
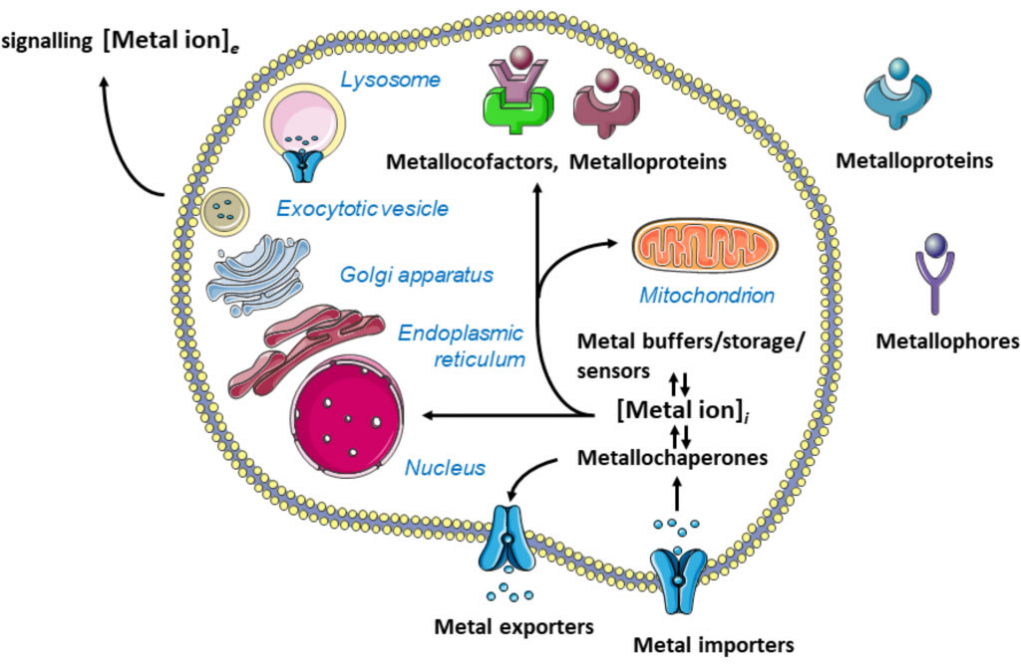
Types of biomolecules and proteins that have evolved to control cellular metal metabolism and homeostasis. Shown are only proteins that are directly involved in binding metal ions. The proteins or metabolites themselves are controlled by many additional biological pathways—some of them metal-dependent—and systemic regulation, e.g. hormones like hepcidin in the case of iron with integration into the physiology of an organism. Each protein has evolved with selectivity for a particular metal ion and with exquisite chemical features adapted to biological function. Despite of this specificity, there is some promiscuity that allows non-essential metal ions to utilize these molecules to gain access to blood, tissues, and cells, and for intracellular redistribution. The schematic figure focuses on a eukaryotic cell and demonstrates the complexity in intracellular metal ion traffic. There is a low molecular weight pool of metal ions designated as [metal ion]*_i_*. Some metal ions are employed in intracellular signalling or are secreted as autocrine or paracrine signalling ions designated as [metal ion]_e_. Eukarya and prokarya differ in their subcellular structures and in the complement of biomolecules and mechanisms they employ. The figure was composed from Servier Medical Art templates (http://smart.servier.com).

An important development for all essential metal ions was the realization how complex their metabolism and control of homeostasis are. Thus, not only do the metal ions control proteins but many proteins also control metal ions. Various types of proteins with special functions are needed for this control (Fig. 2). Specific mechanisms for biological control have evolved for each essential metal ion. Suffice it to say that each metal ion is buffered in a specific range of amounts, related to the buffering capacity, and concentrations, related to the buffering pMe, defined as −log[Me], to minimize interference with the other elements and to generate specificity in its actions. The subject of regulation will not be treated here as it took a rather extensive compendium with 1000 pages in 2014 to provide an overview.^58^ A working knowledge about the regulation of **Na, K, Mg, Ca, Mn, Fe, Cu, Zn** is necessary, however, to understand how non-essential metal ions interact with the systems controlling the essential metal ions. In addition, there is control via specific cofactor synthesis to handle metal ions, not only for heme iron, perhaps as a prerequisite to deal with the required metal ions at low abundance. For control of **Mo**, pterin biosynthesis is required to assemble the molybdopterin cofactor. The biochemistry of **Co** is linked to the corrin cofactor in vitamin B_12_ (cobalamin). The molecular mode of action and the specific biological ligands of **Cr** remain unknown although the first experiments to demonstrate physiological functions date back well over 60 yr.^59^ Chromium had been reasonably well established as being essential and was adopted as such in the nutrition guidelines in many countries.^60^ However, during the last 20 yr a controversy developed whether chromium is nutritionally essential or merely beneficial.^61^ A characteristic feature of chromium biology is the difference in biological activity of two of its valence states. Cr(III) is the form with the positive bioactivity on glucose and lipid metabolism while Cr(VI) in the form of chromate is a carcinogen.^62^ In blood, chromium(III) binds to transferrin, which was called siderophilin at the time this binding was established.^63^ After uptake into cells through receptor-mediated endocytosis, Cr(III) is released from transferrin at the low pH value of endosomes. It has been suggested that Cr(III) then binds to LMWCr (low-molecular-weight chromium-binding substance).^64^ LMWCr was isolated from rabbit liver and characterized with a molecular mass of 1500 Dalton and a composition of glutamic acid/glutamine, glycine, cysteine, and aspartic acid/asparagine and four Cr(III) ions.^65^ LMWCr is an oligopeptide that contains the partial sequence EEEEGDD and is also referred to as chromodulin.^66^ Neither the full sequence of the peptide nor whether it is a cleavage product from a larger protein is known. The composition is similar to material with glucose tolerance factor activity isolated from brewer's yeast, with the notable difference that the latter also contained nicotinic acid.^67^ LMWCr binds Cr(III) cooperatively with an overall formation constant in the order of 10^21^ and can remove Cr(III) from the Cr(III)_2_ transferrin complex. At pH 7.4, it takes over 2 weeks for the reaction to reach equilibrium.^68^ However, at the endosomal pH of 5.5 and in the presence of the transferrin/transferrin receptor complex, the rate of binding is significantly faster.^64^ These investigations demonstrate how a central issue of chromium biochemistry, namely the high kinetic inertness of Cr(III) complexes, can be overcome in appropriate biological coordination environments that make ligand exchange feasible on a biological time scale. The molecular targets of Cr(III) in the cell remain a matter of debate.

With the multitude of molecular functions of metal ions, one wonders whether a hierarchy exists in their redistribution to serve their molecular functions. Iron is distributed into heme, non-heme, and Fe–S proteins. For copper transfer to proteins, a limited number of metallochaperones operate. But for zinc the question of redistribution is mainly unresolved. What are the driving forces for having a higher zinc concentration inside versus outside cells and for redistributing zinc intracellularly? With so many functions and so many proteins with different coordination environments, are all functions of zinc compromised simultaneously or are specific molecular functions primarily affected when zinc supply becomes limiting or competing non-essential metal ions are present?

### A short walk through the periodic table of the non-essential bioelements

Research focuses primarily on the essential elements of life, notwithstanding the question which ones ought to be included in this category. Less attention is paid to the other elements being present in our bodies, perhaps based on the erroneous assumption that non-essential means non-functional.

An exceptional discovery, which amplifies the earlier-mentioned argument about a need to attend to yet other micronutrients in precision nutrition, is that some human gut bacteria utilize tungsten in enzymes that use reactive aldehydes as substrates, implying a detoxifying and protective role.^69^ The discovery demonstrates that we need to know more about elements that are considered non-essential and how we keep our microbiota, for which other elements are essential, nourished. And this metal ecology may even include the microvirota, all the viruses representing the microvirome. With only a total of 20 μg in the human body, tungsten is present at one of the lowest amounts of all detected elements. Its concentrations are at the detection limit in most food and at picomolar concentrations in drinking water. However, there is exposure from the environment. Tungstate leached from, e.g. ammunition is a health hazard due to its toxic actions, in part through a chemical mimicry relating to its congener molybdenum in the periodic table but also relating to its similarity to the phosphate anion.^70^

Another example of the rich biology and biochemistry of presumably non-essential metal ions is titanium. At about 20 mg, the amount of titanium is three orders of magnitude higher than tungsten and higher than at least five essential metal micronutrients, and thus relatively abundant. Its concentration in blood is 2 μM, about as much as that of iodine. It is one of many elements in need to be scrutinized further. It has been discussed that its biological role would be easy to miss.^71,72^ Titanium is bioactive with both positive and negative effects. As with many other elements, titanium metalloproteins such as Ti(IV) transferrin can be made *in vitro*, but their identification *in vivo* remains to be demonstrated.

The necessity to screen for the presence of non-essential elements in living organisms in addition to essential elements with simultaneous, multi-element analyses and to address their functions is becoming quite important. The reason is that new technologies, manufacturing practices, and industrial emissions expose us to a different environment of elements and their compounds, including nanoparticles. We have not been exposed to many of them in the past. They become new bioelements. The pollution of air, soil, and water affects food chains and webs, adding an additional dimension to the normal ecology between biosphere and geosphere. A public science article called ‘the materials bonanza’ discusses that while only one to two human generations ago mineral mining was restricted to a few elements such as Fe, Al, Cu, and Zn, activities now extend to virtually all the elements of the periodic table.^73^ Aside from the economic and political implications, the environmental impact of the newer elements on a per-weight basis in terms of toxicity is far greater than that of the traditionally mined elements. The electronics industry uses rare-earth elements and many transition elements of the second and third rows in the periodic table. One can add the recent shift to lithium ion batteries, which also utilize cobalt in their cathode. Exposure to increased cobalt concentrations is a toxicological hazard.^74^ With 57.4 million tonnes of electronic waste generated in 2021,^75^ one wants to know to which extent we are exposed to some of these elements and how such an exposure changes with time and changes the distribution of elements present in our bodies, and importantly what the functional consequences are. As an example of the additional burden to our environments, the chemical elements used in cell phones will be discussed. Mobile phones contained about 35 chemical elements in 1983 but contain between 65 and 70 elements in smartphones nowadays.^76^ At least two elements, which express toxicity, Be and Pb, have been removed, but in the process of greater functionality many more with proven or unexplored toxicity have been added, leaving the impression that we are ‘jumping from the frying pan into the fire’ to use a common adage. Our knowledge about the functions of these elements when they become bioelements is very limited or even non-existent. We need to understand their uptake and excretion, if any, their biological roles, and how they interact with essential elements. Some of the elements remain in our bodies or accumulate during lifetime. Cadmium is such a biological ‘forever chemical’. Both positive and negative effects must be considered without downplaying the toxicity that is expressed. In the health sciences, many elements are used with significant benefits. Biomedicines fill almost the entire medical periodic table and some of the elements are selected as drugs because of their potency of action and toxicity, e.g. the extremely successful platinum-based anticancer drugs.^77,78^ Arsenic is a constituent of the drug salvarsan (arsphenamine), which was the first effective drug to treat syphilis and trypanosomal disease. Thiomersal contains mercury, also in an organic compound, and is employed as an antifungal and antiseptic preservative, and has been used in vaccines. On the other side, arsenic and mercury contamination of our food continues to be a serious issue to consider and to monitor.^79–82^ The mentioning of these two elements does not imply that there are no other elemental toxicants in our food. Though tetraethyllead was banned as a gasoline additive from 1996 through the issue of the clean air act in the USA, it continued to be sold in some countries until this year. The biological effects of the lead deposited through emission, however, are still seen and will be seen for years to come.^83,84^ Relatively large-scale poisoning of populations with lead and cadmium are well documented.^85,86^ An area of benefit for diagnostic medicine is nuclear medicine, which uses many elements covering a large fraction of the periodic table for imaging, e.g. positron emission tomography metallomics.^87,88^ And then there are implants containing titanium, CoCrMo total hip replacements and prosthetic devices from which metal leaches with biological effects such as sterile inflammation.^89,90^ For many of these elements, it is necessary to explore the negative side effects in terms of specific molecular pathways in more detail. The toxicity of lanthanide (rare-earth elements, all metals) complexes employed in nuclear magnetic resonance imaging is discussed.^91,92^ Lanthanides also exemplify the ambivalence of function. They serve as cofactors of enzymes of some bacteria, and they have been introduced as new, and apparently deemed safe, feed additives to improve animal growth, health, performance, and respective production of animal products.^93,94^

Contemplating the ramification of the new exposures in this way, the quest for new materials, the materials bonanza, is destined to become a bonanza for metallomics.

### Selectivity and promiscuity

A certain duality exists in biology regarding the chemical similarity of the elements in each group of the periodic table. On one side, one discerns a push of biology to acquire additional elements in the same group for expanding functions, e.g. Se and W extending the biochemistry of S and Mo, respectively. On the other side, organisms must protect themselves against the chemical mimicry of similar elements. For instance, cadmium and mercury have much higher affinity for coordination environment with sulphur donor ligands than zinc and therefore compete at lower concentrations. One way of protection is to ascertain that enough of the essential element is present for its function. Conditions of deficiency pose a risk of accumulating a chemically similar element expressing toxicity. For example, more cadmium is taken up when zinc or iron are deficient. Chemical similarity, while also extending to elements with similar ionic radii and charges outside each group, can be exploited in pharmacology, e.g. Sr for Ca used in treating osteoporosis or Rb for K used in myocardial perfusion imaging.

While there is selectivity, there is also promiscuity. The selectivity of coordination environments in proteins is not absolute. Elements other than the native ones—assuming one knows with certainty which ones are native—can be incorporated into the active sites of metalloenzymes *in vitro* and result in catalytically competent enzymes. This approach is very successful for investigating zinc, which is ‘invisible’ for most spectroscopies. Substitution of zinc by other metal ions that are amenable to spectroscopic studies has provided a wealth of information about how the catalytic metal ion functions in zinc enzymes.^95^ When comparing the catalytic competency of different metal ions, it was noted that functions of a metalloenzyme are not necessarily optimized with its native metal ion(s).^96^ The question which metal ion is employed depends on the availability of the metal ion from the environment and the systems that control it in the organism and in its cells. The metal homeostatic control of a cell can overrule the metal ion selectivity of a metalloprotein. Environmental, inorganic, and biochemical factors determine metalation specificity or promiscuity. If, on the other side, a specific element needs to be employed, e.g. if zinc does not serve the purpose, then additional proteins such as transporters and metallochaperones perform the specific metalation, e.g. for nickel ureases or cobalt nitrile hydrolases.^96^ Experimental exposures have shown that organisms can employ metal ions other than the native ones. Cobalt enzymes form when cobalt is offered instead of zinc to *Salmonella Typhimurium*.^97^ Our knowledge of the composition of metal sites in native metalloproteins is incomplete. Does a zinc metalloprotein *in vivo* always contain 100% zinc or does it contain small amounts of other metal ions, and if it does, does the composition vary depending on availability? Addressing these questions by analysing the metal contents of purified proteins has been inconclusive as it cannot be ruled out that metal scrambling occurs during the occasionally harsh conditions of isolation. The functional consequences of metal exchange or substitution can be significant, ranging from entirely inhibiting an enzymatic activity, e.g. when a redox-inert metal such as zinc replaces a redox-active metal ion such as copper, to changed catalytic properties or altered substrate specificity. Could switching metal ions even be used for modulating protein function? For some classes of enzymes, it is not certain which metal ion is the native one. For technical reasons, many 3D structures of phosphatases and related enzymes have been solved with manganese although the native metal is expected to be magnesium. Furthermore, proteins that are not saturated with metal ions such as transferrin or metallothionein provide an opportunity for other metal ions to hitchhike. Notably, this exegesis also points at the limitations of experimentation. Heterologous expression of metalloproteins is common. However, the organism employed for expression has different systems for metal control. In investigations with cultured cells and organoids, all the extracellular selectivity filters for metal ions are missing. The growth media used do not reflect the body's composition, and mineral availability is mostly restricted to those present in the serum if full media are used. Experiments with serum-free media are even more problematic when they rely on unknown or poorly characterized sources of micronutrients. This lack of control is a significant factor of variability in experimental outcomes and the ensuing expected coordination promiscuity under these conditions finds almost no attention. In a way, it reflects the pinch points, on an organismal scale, in our limited knowledge about the trace element composition of the food and in dealing with the consequences for health.

## Future

### The field of metallomics

Another citation from the same article is also highly relevant 70 years later:^6^

‘The problem of trace elements in living systems cannot be assigned to any one particular field, …Oddly enough, much attention has been devoted to these complex molecules…, but their interaction with simpler inorganic compounds derived directly from the soil has been unduly disregarded’.

The first sentence, assignment to one field, refers to the fact that bioelements are addressed in many different scientific disciplines, e.g. nutrition as micronutrients, pharmacology as metallodrugs, toxicology as toxicants, often independently and from different perspectives. The second sentence, the transformation of inorganic compounds into bioinorganic compounds remains addressed insufficiently as illustrated in the flow of elements with at least four steps:

- available form of the elements in water, air, or soil (leaching of elements from minerals aided by bacteria);
- metallophores employed for the binding of some metals to increase the chances of acquiring low abundance metals from the environment;
- element taken up and becoming a bioelement; and(iv)  (re)mineralization of the elements aided by bacteria when the organism dies.

This topic of the interaction between the biosphere and geosphere already gained prominence in the intriguing observation that metalloproteomes harbour information about evolutionary changes that occurred in geochemistry.^98^ Addressing the interface between biology and the purely inorganic world is becoming even more important with our recent exposure to new materials. ‘Green metallomics’ could offer a synthesis of knowledge. Metallomics investigations ought to include studies of chemical structures and transformations, including the entire area of the chemistry and physics of nanomaterials.

Non-biological metallomics includes the chemical forms in which the metal ions are present in the diet. Biological metallomics then begins with which metal complexes present are transported into the intestinal cells, how the complexes change their ligands during transport and how they adopt yet different coordination environments in blood, cells, and subcellular compartments before finding their final destinations. For example, the nature of the ligands of zinc(II) ions other than those in proteins are rarely defined in investigations and the coordination and its changes is expected to be rather complex and dynamic.^99^

The crux of the matter is that scientists become ever more focused on single disciplines or even subdisciplines as reflected in the plethora of scientific societies, conferences, and journals. Biological inorganic chemistry—with the permutations of the name of the field and changes of its emphasis: bioinorganic chemistry or inorganic biochemistry—focuses on interactions of bioelements, mostly biometals, with biological molecules, mostly proteins. In contrast, metallomics takes a holistic, systems biology approach to metal biology, and explores static as well as dynamic aspects such as the spatiotemporal distribution of metal ions in cells and entire organisms, and their interactions with the environment. Metallomics can serve as an umbrella for many disciplines. It seeks integration with other ‘omics fields and considers the connections of ‘omes, the roles of metalloproteomes, which are the most widely studied, but also metallolipidomes, metalloglycomes, metallogenomes, metallotranscriptomes, and metallometabolomes, resulting in multi-omics approaches or characterization of multi-omes such as integrative studies of the urinary metallome and the blood metabolome.^2,100^ Like other ‘omics disciplines, it has several forms of inquiries and approaches:

- structural metallomics,
- functional metallomics,
- analytical metallomics,
- computational metallomics, and
- comparative metallomics.

Comparative metallomics developed from comparative genomics as a means of obtaining information on the evolution of function and utilization of metal ions. It encompasses aspects of all the other forms of inquiry in metallomics.^101^ An interesting outcome is that, while iron and zinc are employed almost universally by organisms, the usage of the other transition metals varies significantly in the three domains of life.^102^ ‘Metametallomics’, in analogy to metagenomics, comprises investigations of mixed communities of organisms. It can include multiple dimensions such as genetic heterogeneity, disease states, among others. In addition to this subdivision in terms of methodologies and combination of fields such as pharmacometallomics, toxicometallomics, nutrimetallomics, or ecometallomics, many new compound words have been suggested to emphasize the role of metallomics in a range of disciplines and applications where metals ions are important.^103^ Without trying to be comprehensive with more combinations, one should follow the simple advice given in an article that the ‘ome or ‘omics word should be easy to understand and to say.^104^ New words for hybrid fields do not necessarily integrate and can detract from focusing on the essence of metallomics, which needs to be strengthened rather than weakened by too many subfields. Metallomics should be seen as the platform for research subjects in basic sciences, life sciences, and mission-oriented sciences.^105^

Specific omes/omics can be defined either for individual metals or specific areas. Terms such as zinc metallome, zinc metallomics, and zinc proteome all have found a place and so has metallomics when referring to protein families that involve several metal ions, e.g. metallothionein metallomics.^32,33,106^ Another area of inquiry concerns the metalloproteomes of metals that are thought to be non-essential for humans. Exposure to nickel can lead to allergy or carcinogenesis, making the exploration of the nickel proteome worthwhile.^107^ In principle, a metallomics approach can be applied to any metal ion, and it has already been applied to many. Metallomes referring to tissues, fluids, or diseases and a combination thereof are also established. Selected examples are the serum metallome investigated in pregnant women to examine a correlation with malformations in the brain,^108^ the urinary metallome in breast cancer patients,^109^ or the placental metallome in gestational diabetes.^110^ All these endeavours show the importance of multi-metal screening. Additional metallomics approaches involve the roles of a specific metal ion or several metal ions in a specific disease, such as the human copper cancer proteome^111^ or the human lung cancer metallome as investigated with bronchoalveolar lavage fluid and discussed in the context of concomitantly acquired metal profiles in serum and urine.^112^ Such investigations demonstrate the feasibility of biomarker discovery for disease or for exposure with a non-invasive screening approach that detects perturbations of the metabolism of essential elements in inflammatory and other diseases processes or in exposures to disease-causing metal contaminants. It should be a fruitful area for collaborations between analytical and clinical chemists. And as a final example, metallomics in the field of biomineralization has been called mineralomics.^113^

### Present challenges in analytical metallomics

This special issue ‘Analytical Tools in Metallomics’ is timely to address the challenges that come with the scientific questions raised in this article. To name a few: Despite of all the advances, detection limits continue to be an issue, e.g. in single cell metallomics. Technology has been developed with stunning results for 3D metal imaging in biology.^114,115^ In addition to spatial resolution in 1D, 2D, and 3D applications, the next area of inquiry into the dynamics of metal metabolism will require temporal resolution. Metal imaging shows uneven distribution of metal ions in organisms, tissues, and cells and is awaiting further applications employing human tissues. Imaging through different scales, using many spectroscopies and microscopies throughout the electromagnetic spectrum and merging the results will continue to make significant contributions.^116^ Inquiries with multi-omics, multimodal, and multiplexing approaches promise to deliver a wealth of new information. In addition to targeted metal analyses, multi-elemental profiles obtained with fast acquisition elemental time-of-flight MS offer untargeted approaches that will be necessary to screen for changes of bioelements in health and disease. New workflows, computational approaches, and methods of image analyses need to support these developments and the fusion and integration of multi-dimensional datasets and strategies. It will require deep learning, artificial intelligence, and machine learning to cope with the massive amount of data and to aid in their interpretation.^117^ Speciation analysis has made great strides in the analyses of arsenic, selenium, and even tellurium compounds^118^ but needs to be further developed for non-protein complexes of metal ions. And one area not touched upon here is the determination of the natural stable isotope composition and isotope fractionation for biomarker discovery, studying disease processes, and mapping metal metabolic pathways, as, e.g. applied to non-invasive investigations in blood^119^ or in urine for pancreatic ductal adenocarcinoma.^120^

### Teaching and educating in metallomics

A challenge in contemporary education is to tailor integrative and interdisciplinary courses to students in different fields in order to capture the fast-growing knowledge in the life sciences and overcome narrow specialization. Teaching the chemistry of the bioelements in biology is virtually non-existent. A case for incorporating elemental biology with its methodological aspects into both general and specific education has been made.^121^ Furthermore, teaching of some important areas is also largely non-existent in life sciences curricula. It includes such complex areas as sustainability, economy, disease prevention, politics, risk assessments, recycling, and all their interrelations in the context of supporting our survival in a global setting.

## Conclusions

Since perturbation of metal and element metabolism is a significant aspect of how disease is caused and develops, future metallomics investigations will contribute to improving human health and treating diseases. Additional ventures into the uncharted territory of the bioactivity of so-called non-essential elements will be important. Addressing new exposures from ongoing anthropogenic activities that change the ecology of our environment will be important. A sound foundation for the field of metallomics will require bringing many disciplines together towards an understanding of the periodic table of the bioelements and revisiting what apparently is considered resolved by many: the number of essential elements for humans and for life in general.^122^

Angela Gronenborn, in a review titled ‘integrated multidisciplinarity in the natural sciences’ makes the point that in order to capture the features of complex biological systems, which work

‘at different scales and levels of organization … individual methods and models comprise abstractions from an idealization of nature, and only the integration of multiple models, methods, and representations provides a means to reach more accurate results than relying on any single approach. …any one methodological approach alone results in a partiality of representation … interdisciplinary research leads to breakthroughs in science’.^123^

One would hope that these statements will serve as a mantra for those practicing metallomics. But most importantly, the call is out for other life scientists to consider metallomics and elemental biology as essential parts of their investigations in cellular and molecular biology.

## Data Availability

No new data were generated or analysed in support of this research.
